# Physical activity inversely associated with the presence of depression among urban adolescents in regional China

**DOI:** 10.1186/1471-2458-9-148

**Published:** 2009-05-20

**Authors:** Xin Hong, JieQuan Li, Fei Xu, Lap Ah Tse, YaQiong Liang, ZhiYong Wang, Ignatius Tak-sun Yu, Sian Griffiths

**Affiliations:** 1 Nanjing Municipal Center for Disease Control & Prevention, Nanjing, PR China; 2 Department of Community and Family Medicine, The Chinese University of Hong Kong, Hong Kong, PR China; 3 Center for Occupational and Environmental Health Studies, School of Public Health, The Chinese University of Hong Kong, Hong Kong, PR China; 4 School of Public Health, The Chinese University of Hong Kong, Hong Kong, PR China

## Abstract

**Background:**

An inverse relationship between physical activity (PA) and depression among adolescents has been reported in developed communities without consideration of sedentary behaviors (SB, including sitting for course study, viewing TV, and sleeping). We explored the association between recreational PA time (hr/wk) and depression after adjustment with SB and other possible confounders among Chinese adolescents.

**Methods:**

A population-based cross-sectional study was conducted in Nanjing municipality of China in 2004 using a multi-stage cluster sampling approach. A total of 72 classes were randomly selected from 24 urban junior high schools and all students completed the structured questionnaire. Adolescent depression was examined by the Children's Depression Inventory (CDI) of Chinese version with cutoff point value of 20 or above as the presence of depression. Recreational PA time was measured by a question on weekly hours of PA outside of school. Descriptive statistics, multivariate logistic and linear regression models were used in analysis.

**Results:**

The overall prevalence of depression was 15.7% (95%CI: 14.3%, 17.1%) among 2,444 eligible participants. It was found that physical activity was negatively associated with depression. After adjustment for sedentary behaviors and other potential confounders, participants who spent 1–7 hr/wk, 8–14 hr/wk and 15+ hr/wk for recreational PA, respectively, had odds ratios of 0.70 (95% CI = 0.57, 0.86), 0.68 (95% CI = 0.53, 0.88) and 0.66 (95% CI = 0.50, 0.87) for likelihood of being depressive, compared to their counterparts who spent 0–0.9 hr/wk for PA. This inverse relationship between PA time and depression remained statistically significant by gender and grade.

**Conclusion:**

This study, conducted among Chinese adolescents, strengthened the evidence that physical activity was inversely associated with depression. Our study has important implications for health officers and public health professionals to pay much attention to the relationship between physical activity and depression in Mainland China.

## Background

Depression is a major public health problem worldwide, and it is predicted to be the second leading cause of disability by 2020, immediately behind cardiovascular diseases [1]. The prevalence of depression among adolescents was estimated to be between 0.4% and 8% in 2006 [2]. Evidence from both cross-sectional [3-14] and longitudinal studies [15-18] among Western adolescents consistently showed an inverse relationship between physical activity (PA) and depression. However, duration of PA and sedentary behaviors (SB, including sitting for course study, viewing TV, and sleeping) were seldom considered in these studies. Moreover, one clinical trial found there was a dose-response relation between the exercise amount and reduction in depressive symptoms [19].

China, the most populous country in the world, is undergoing a rapid economic and lifestyle transition from traditional agricultural society to an industrialized community. The employment market is becoming more and more competitive than ever before. Meanwhile, Chinese students typically spend more time on academic studies and less time in physical activity compared to their counterparts in Western countries [20]. Thus, it is of unique importance to explore the associations between PA and depression among adolescents in the current context of Mainland China, with consideration of SB.

We hypothesized that PA time (hr/wk) was inversely associated with depression among Chinese adolescents after taking into consideration of SB and other possible confounders. We conducted a large-scale survey among urban junior high school students between September and November of 2004 in Nanjing of China to examine the hypothesis.

## Methods

### Sample selection and participants

This population-based cross-sectional study named Nanjing High School Students' Health Survey (NSHS) was conducted in Nanjing municipality. Nanjing is located in eastern China, with a total population of about 6.0 million in 13 administrative units (11 urban districts and 2 rural counties) in 2004. In Mainland China, the school system consists of four strata: kindergarten, primary school (grade 1–6), high school (junior: grade 7–9, senior: grade 10–12), and college/university.

The class-based samples were selected using a multi-stage cluster sampling method. First, we estimated the number of participants based on the available rate of depression, 11.5%, among urban junior high school adolescents; thus, the total number of participants required was estimated to be 2,500. Second, according to the total number of grade-specific enrolled students and urban junior high schools by district in 2003, we calculated the sample size and sampling proportion for each urban district. Third, based on the estimation of approximate 40 students in one class and the sample size, the number of participated classes was calculated for each urban district. Fourth, taking the principle that one class would be randomly selected from each grade in each selected school, the number of participated schools was calculated; then, we randomly selected schools within each urban district. Finally, each class was randomly selected from each grade within each chosen junior high school. This resulted in a total of 72 classes from 24 junior high schools. All students from those selected classes were invited to participate in this study.

This study was approved by the academic and ethical committee of Nanjing Municipal Center for Disease Control and Prevention in accordance with the internationally agreed ethical principles for medical research involving human subjects (WORLD MEDICAL ASSOCIATION DECLARATION OF HELSINKI).

### Questionnaire

After informed consents were obtained, the students were asked to complete a self-administered anonymized questionnaire. The questionnaire included general information, such as age, gender, grade, class, body weight and height, status of parents' job and education, family structure; unintentional injures, health risk behaviors (experience of smoking and drinking), and some specific questions regarding time engaged in recreational physical activity (PA time), time spent on viewing TV/video (TV time), time spent in sitting for course study (study time), and time spent on sleeping (sleep time). The 27 items of the Children's Depression Inventory (CDI) were also organized in the questionnaire [21].

### Definitions

#### Depression

In this study, CDI was used as the instrument to screen depression in junior high school students [21]. CDI is a validated questionnaire originally developed by Kovacs and has been widely used to measure depression for children and adolescents in epidemiological studies [22-27]. It contains 27-items with a self-rating scale ranging from 0 to 2 (e.g., What do you think of yourself? 0 = I like myself; 1 = I do not like myself; 2 = I hate myself) that yield total scores from 0 (no indication of depression) to 54 (high depressive tendencies), where higher scores reflect severity of depression.

The Chinese version of CDI has also been validated, with satisfied internal consistency (Cronbach's α = 0.85–0.89) and test-retest reliability (r = 0.75–0.85) with cut-off point value of 20 [28-30]. In this study, the CDI Cronbach's α was 0.85 showing an acceptable validity. The term of "depression" based on CDI refers to an epidemiological idea not a clinical diagnosis in this study. That is to say, it definitely means that all adolescents with CDI value of 20 or above were at elevated risk of being depressive, which should not be interpreted as a clinical diagnosis.

#### Time spent on physical activity and sedentary behaviors

Each student reported time spent in doing recreational physical activity, viewing TV, sitting for course study, and sleeping through the survey question "How much time (in minutes) did you spend (doing recreational physical activity/watching TV/sitting for course study/sleeping at night or during the day) on a typical weekday in the past two weeks?" The same question was repeated for a typical weekend day. Then, PA Time, TV Time, study Time and sleep time were, respectively, calculated as the average time spent in recreational physical activity, viewing TV, sitting for course study, and sleeping per week. Sedentary behaviors refer to sitting for viewing TV, course study and sleeping in this study. The PA and sedentary behaviors were mainly based on international physical activity questionnaire (IPAQ) with recall period extended from last week to last 2 weeks [31]. PA time was treated as either a continuous (hr/wk) or categorical (grouped as: 0–0.9, 1–7, 8–14 and 15+ hr/wk) variable to examine its association with depression, while TV time, Study time, and Sleep time were categorized into three levels by tertiles: low, middle and high. Time spent on sedentary behaviors included TV time, Study time, and Sleep time.

#### Overweight

Body weight (kg) and height (cm) were self-reported by students. Body mass index (BMI) was calculated by dividing weight (kg) by the square of height (m^2^). Overweight was defined as a BMI ≥ 85 percentile value for age- and sex-specific reference data according to the recommendation for Chinese adolescents by the Group of China Obesity Task Force [32]. In China, all students are required to have their body weight and height measured in the commencement month (generally September) of each academic year. This was one of the most important reasons for us to conduct this study between September and November: to minimize potential recall errors for self-reported body weight and height.

#### Smoking behavior and alcohol consumption

A student who ever smoked at least one entire cigarette one time was defined as having an experience of smoking behavior. Having an experience of alcohol consumption referred to ever drinking at least 50 gram distilled spirit (at least 40% alcohol) or one bottle of (≥ 600 ml) beer.

#### Unintentional injuries

Participants were asked to report the frequencies and kinds of unintentional injuries happened in the past year. Unintentional injuries covered car accidents, bone fractures, falls, knife punctures and other injuries

#### Socioeconomic status (SES)

Parental educational attainments were categorized into four groups: Junior high school or less, Senior high school, Undergraduate, and Graduate, while parental job statuses were classified as both employed, single employed, and both unemployed. Family structure was grouped into core family (both parents), single parent family and others.

#### Data management and analysis

Chi-square test was used to examine the association between time spent on PA (by category) and conventional potential confounders of depression. We calculated the prevalence of depression in each population group. Association of PA category (1–7 hr/wk, 8–14 hr/wk and 15+ hr/wk, respectively) with depression was analyzed via both multivariate logistic and linear regression models with adjustment for age, gender, school grade, BMI, TV time, study time, sleep time, smoking behavior, alcohol consumption, unintentional injuries, parents' educational attainments, parents' job statuses and family structure.

Data were double-entered and cleaned with EpiData (Version 3.0, 2005; the Epidata Association; Odense Denmark), and managed and analyzed using SPSS (Version 13.0, 2001; SPSS Chicago, Illinois, USA).

## Results

### Demographic characteristics of the participants

The total number of respondents was of 2,444 in the survey, with a response rate of 93.4%. Of total participants, 31.4% (768), 33.9% (829) and 34.7% (847) were, respectively, from grade 7, 8 and 9, with 48.3% (1180) of boys and 51.7% (1264) of girls. The mean age was 13.85 ± 1.04 years old, and there was no significant difference between boys (13.86 ± 1.05) and girls (13.84 ± 1.03) (p = 0.590). No significant differences were observed between the respondents and non-respondents in terms of age, gender, and grade.

### Factors associated with time spent on physical activity

The mean hours spent on physical activity was 9.8 ± 7.8 hr/wk in this sample population, while 43.1% of students spent 1–7 hr/wk on physical activity. As shown in Table 1, PA time was significantly associated with gender, school grade, parental educational attainments, family structure, alcohol consumption, and time spent on SB, with exception for BMI, smoking behavior and unintentional injuries. Participants in inactive group had more alcohol consumption experiences, and spent more time on studying and watching TV relative to their active counterparts.

**Table 1:** Associations of physical activity time categories with potential confounders among urban high-school students in Nanjing, China

	Physical Activity Time (hr/wk) (n, %)					
			
Characteristics	0–0.9	1–7	8–14	15 +	*χ*^2^	p*
**Gende**r						
Girls	286 (54.8)	590 (56.0)	243 (49.2)	145 (38.7)	36.659	< 0.001
Boys	236 (45.2)	463 (44.0)	251 (50.8)	230 (61.3)		
**School Grade**						
7	132 (25.3)	337 (32.0)	159 (32.2)	140 (37.3)	24.219	< 0.001
8	173 (33.1)	350 (33.2)	176 (35.6)	130 (34.7)		
9	217(41.6)	366 (34.8)	159 (32.2)	105 (28.0)		
**Body Mass Index**						
Non-overweight	450 (86.2)	933 (88.6)	444 (89.9)	341 (90.9)	6.588	0.361
Overweight	47 (9.0)	77 (7.3)	35 (7.1)	24 (6.4)		
Obesity	25 (4.8)	43 (4.1)	15 (3.0)	10 (2.7)		
**Father education**						
Junior high school or less	180 (34.5)	366 (34.8)	142 (28.7)	140 (37.3)	31.108	< 0.001
Senior high school	222 (42.5)	454 (43.1)	205 (41.5)	175 (46.7)		
Undergraduate	102 (19.5)	195 (18.5)	125 (25.3)	42 (11.2)		
Graduate	18 (3.4)	38 (3.6)	22 (4.5)	18 (4.8)		
**Mother education**						
Junior high school or less	242 (46.4)	475 (45.1)	188 (38.1)	135 (36.0)	50.965	< 0.001
Senior high school	222 (42.5)	434 (41.2	189 (38.3)	155 (41.3)		
Undergraduate	52 (10.0)	130 (12.3)	110 (22.3)	77 (20.5)		
Graduate	6 (1.1)	14 (1.3)	7 (1.4)	8 (2.1)		
**Family structure**						
Both parents	480 (92.0)	925 (87.8)	413 (83.6)	320 (85.3)	24.745	< 0.001
Single parent	24 (4.6)	88 (8.4)	63 (12.8)	43 (11.5)		
Others	18 (3.4)	40 (3.8)	18 (3.6)	12 (3.2)		
**Study time**†						
Low	205 (39.3)	314 (29.8)	171 (34.6)	141 (37.6)	28.148	< 0.001
Middle	141 (27.0)	351 (33.3)	175 (35.4)	132 (35.2)		
High	176 (33.7)	388 (36.8)	148 (30.0)	102 (27.2)		
**Sleep time**†						
Low	200 (38.3)	348 (33.0)	138 (27.9)	112 (29.9)	22.317	0.001
Middle	144 (27.6)	366 (34.8)	173 (35.0)	113 (30.1)		
High	178 (34.1)	339 (32.2)	183 (37.0)	150 (40.0)		
**TV time**†						
Low	148 (28.4)	338 (32.1)	170 (34.4)	150 (40.0)	41.527	< 0.001
Middle	150 (28.7)	313 (29.7)	182 (36.8)	125 (33.3)		
High	224 (42.9)	402 (38.2)	142 (28.7)	100 (26.7)		
**Alcohol consumption**						
Non	380 (72.8)	715 (67.9)	394 (79.8)	285 (76.0)	26.589	< 0.001
Yes	142 (27.2)	338 (32.1)	100 (20.2)	90 (24.0)		
**Smoking behavior**						
Non	491 (94.1)	989 (93.9)	476 (96.4)	360 (96.0)	5.701	0.127
Yes	31 (5.9)	64 (6.1)	18 (3.6)	15 (4.0)		
**Unintentional injuries**						
Non	348 (66.7)	666 (63.2)	311 (63.0)	224 (59.7)	4.624	0.201
Yes	174 (33.3)	387 (36.8)	183 (37.0)	151 (40.3)		
**Overall**	522 (21.4)	1053 (43.1)	494 (20.2)	375 (15.3)	-	-

n = number of participants within subgroup; % = Percentages across column.

* p-value between sub-groups of each variable.

† Study time, sleep time, and TV time were classified into tertiles, separately.

### Depression and risk factors

The mean value of CDI score was 11.62 ± 7.49 for all the participants, being significantly higher among boys than that among girls (12.00 ± 7.70 *vs*. 11.22 ± 7.26, t = 2.56, p = 0.01).

Based on the epidemiological definition of "depression" in this study, the overall prevalence of depression was 15.7% (95%CI: 14.3%, 17.1%) among these students. The depression prevalence was positively associated with obesity (adj. OR = 1.43, 95% CI = 1.01, 2.02), parental unemployed status (adj. OR = 1.60, 95% CI = 1.02, 2.51), other family structure (adj. OR = 2.39, 95% CI = 1.35, 4.29), alcohol consumption (adj. OR = 1.97, 95% CI = 1.55, 2.52), smoking experience (adj. OR = 2.69, 95% CI = 1.80, 4.03), and unintentional injuries (adj. OR = 1.45, 95% CI = 1.34, 1.86), but negatively associated with long sleep time (adj. OR = 0.63, 95% CI = 0.47, 0.85) (Table 2).

**Table 2:** Prevalence of depression (n and %) and its association with gender, grade, BMI, SES, SB time, alcohol consumption, smoking behavior, and unintentional injuries among urban high-school students in Nanjing, China

	Prevalence n (%)		
		
	Depression	Non-depression	Adj. Odds ratio (95% CI)**
**Gender**			
Girls	172 (13.6)	1092 (86.4)	1
Boys	212 (18.0)	968 (82.0)	1.04 (0.79, 1.38)
**School Grade**			
7	102 (13.3)	666 (86.7)	1
8	145 (17.5)	684 (82.5)	1.06 (0.77, 1.45)
9	137 (16.2)	710 (83.8)	0.84 (0.61, 1.17)
**Body Mass Index**			
Non-overweight	329 (15.2)	1839 (84.8)	1
Overweight	35 (19.1)	148 (80.9)	1.23 (0.97, 1.56)
Obesity	20 (21.5)	73 (78.5)	1.43 (1.01, 2.02)
**Parental job statuses**			
Both employed	256 (14.1)	1564 (85.9)	1
Father unemployed	24 (24.0)	76 (76.0)	1.69 (0.96, 2.97)
Mother unemployed	66 (18.4)	292 (81.6)	1.25 (0.89, 1.77)
Both unemployed	38 (22.8)	128 (77.2)	1.60 (1.02, 2.51)
**Family structure**			
Both parents	320 (15.0)	1818 (85.0)	1
Single parent	40 (18.3)	178 (81.7)	1.11 (0.72, 1.70)
Others	24 (27.3)	64 (72.7)	2.39 (1.35, 4.29)
**Study time**†			
Low	120 (14.4)	711 (85.6)	1
Middle	128 (16.0)	671 (84.0)	1.03 (0.77, 1.38)
High	136 (16.7)	678 (83.3)	1.12 (0.84, 1.49)
**Sleep time**†			
Low	155 (19.4)	643 (80.6)	1
Middle	120 (15.1)	676 (84.9)	0.77 (0.58, 1.02)
High	109 (12.8)	741 (87.2)	0.63 (0.47, 0.85)
**TV time**†			
Low	128 (15.9)	678 (84.1)	1
Middle	99 (12.9)	671 (87.1)	0.84 (0.62, 1.13)
High	157 (18.1)	711 (81.9)	1.27 (0.94, 1.67)
**Alcohol consumption**			
Non	216 (12.2)	1558 (87.8)	1
Yes	168 (25.1)	502 (75.9)	1.97 (1.55, 2.52)
**Smoking behavior**			
Non	332 (14.3)	1984 (85.7)	1
Yes	52 (40.5)	76 (59.5)	2.69 (1.80, 4.03)
**Unintentional injuries**			
Non	207 (13.4)	1342 (86.6)	1
Yes	177 (19.4)	718 (80.2)	1.45 (1.34, 1.86)
**Overall**	384 (15.7)	2060 (84.3)	-

n = number of participants within subgroup; % = Percentages across row.

** odds ratios adjusted for age, gender, school grade, BMI, TV time, study time, sleep time, smoking behavior, alcohol consumption, unintentional injuries, parents' educational attainments, parents' job statuses, family structure.

† Study time, sleep time, and TV time were classified into tertiles, separately.

### Relationship between depression and physical activity time

We observed a significantly negative association between PA time and depression among participants (Table 3). Compared to the inactive group (0–0.9 hours PA time per week), active students who spent at least one hour time per week in physical activity were at significantly lower (<30%) risk of being depressive, and they had the OR (95%CI) for being depressive from 0.63 (95% CI = 0.54, 0.74), 0.58 (95% CI = 0.48, 0.71) to 0.53 (95% CI = 0.43, 0.66) with PA time increasing from 1–7 hr/wk, 8–14 hr/wk to 15+ hr/wk, respectively.

**Table 3:** Prevalence of depression (n and %) and its association with physical activity time by gender and grade among junior high school students in Nanjing, China

	Prevalence n (%)			
			
PA category (hr/wk)	Depression	Non-depression	Unadjusted Odds ratio (95% CI)*	Adjusted Odds ratio (95% CI)**
**Total**				

0–0.9	120 (23.0)	402 (77.0)	1	1

1–7	147 (14.0)	906 (86.0)	0.63 (0.54, 0.74)	0.70 (0.57, 0.86)

8–14	68 (13.8)	426 (86.2)	0.58 (0.48, 0.71)	0.68 (0.53, 0.88)

15+	49 (13.1)	326 (86.9)	0.53 (0.43, 0.66)	0.66 (0.50, 0.87)

**Gender**				
Girls				
0–0.9	62 (21.7)	224 (78.3)	1	1
1–7	63 (10.7)	527 (89.3)	0.42 (0.29, 0.62)	0.56 (0.27, 0.64)
8–14	32 (13.2)	211 (86.8)	0.55 (0.34, 0.87)	0.65 (0.40, 1.08)
15+	15 (10.3)	130 (89.7)	0.42 (0.23, 0.76)	0.35 (0.17, 0.69)
Boys				
0–0.9	58 (24.6)	178 (75.4)	1	1
1–7	84 (18.1)	379 (81.9)	0.68 (0.46, 0.99)	0.74 (0.48, 1.13)
8–14	36 (14.3)	215 (85.7)	0.51 (0.32, 0.82)	0.54 (0.32, 0.91)
15+	34 (14.8)	196 (85.2)	0.54 (0.34, 0.87)	0.56 (0.33, 0.96)
**School Grade**				
7				
0–0.9	25 (18.9)	107 (81.1)	1	1
1–7	45 (13.4)	292 (86.6)	0.66 (0.39, 1.13)	0.73 (0.49, 0.98)
8–14	14 (8.8)	145 (91.2)	0.41 (0.21, 0.83)	0.50 (0.28, 0.88)
15+	18 (12.9)	122 (87.1)	0.63 (0.33, 1.22)	0.59 (0.28, 1.26)
8				
0–0.9	41 (23.7)	132 (76.3)	1	1
1–7	51 (14.6)	299 (85.4)	0.55 (0.35, 0.87)	0.61 (0.31, 0.85)
8–14	30 (17.0)	146 (83.0)	0.66 (0.39, 1.12)	0.60 (0.33, 1.08)
15+	23 (17.7)	107 (82.3)	0.69 (0.39, 1.23)	0.49 (0.26, 0.96)
9				
0–0.9	54 (24.9)	163 (75.1)	1	1
1–7	51 (13.9)	315 (86.1)	0.49 (0.32, 0.75)	0.53 (0.33, 0.85)
8–14	24 (15.1)	135 (84.9)	0.54 (0.32, 0.91)	0.58 (0.32, 1.04)
15+	8 (7.6)	97 (92.4)	0.25 (0.11, 0.55)	0.24 (0.10, 0.58)

n = number of participants within subgroup; % = Percentages across row.

using "0–0.9 hr/wk" as the reference group.

*odds ratio calculated via univariate logistic regression model.

**odds ratios calculated via multivariate logistic regression model with adjustment for age, gender, school grade, BMI, TV time, study time, sleep time, smoking behavior, alcohol consumption, unintentional injuries, parents educational attainments, parents' job statuses, family structure.

After adjustment for age, gender, school grade, BMI, TV time, study time, sleeping time, smoking behavior, alcohol consumption, unintentional injuries, parents' educational attainments, parents' job statuses, and family structure, active participants were less likely to be depressive relative to their inactive counterparts. However, there was no difference in PA-depression association among three subgroups of active subjects.

To further explore the impact of physical activity on depression, we conducted linear regression analysis with CDI score and physical activity time as continuous variables (Table 4). Both univariate and multivariate linear regression analysis showed that PA time was also negatively associated with CDI score, which can strengthen the finding from logistic regression analysis.

**Table 4:** The association between physical activity time and CDI value* among urban junior high-school students in Nanjing, China, using linear regression models

		**Gender**		**School Grade**		
			
	Total	Girls	Boys	7	8	9
**Model 1^#^**						
B	-0.033	-0.051	-0.029	-0.015	-0.027	-0.057
SE	0.005	0.010	0.006	0.009	0.013	0.021
R^2^	0.005	0.007	0.006	0.002	0.002	0.009
p-value	0.000	0.000	0.000	0.101	0.045	0.005
DF	1	1	1	1	1	1
**Model 2^§^**						
B	-0.030	-0.046	-0.024	-0.011	-0.024	-0.050
SE	0.005	0.015	0.006	0.008	0.012	0.010
R^2^	0.011	0.013	0.008	0.006	0.009	0.012
p-value	0.000	0.002	0.000	0.186	0.040	0.000
DF	15	14	14	14	14	14

*Time spent in physical activity and CDI were both considered as continuous variables, with CDI as the dependent variable.

B: unstandardized coefficient; SE: standard error.

^# ^Model 1: regression coefficient calculated via univariate linear regression model.

^§ ^Model 2: regression coefficient calculated via multivariate linear regression model with adjustment for age, gender, school grade, BMI, TV time, study time, sleep time, smoking behavior, alcohol consumption, unintentional injuries, parents' educational attainments, parents' job statuses, and family structure.

## Discussion

Using the unique definition of depression in this study, we observed a higher prevalence (15.7%) of depression among Chinese urban adolescents than those reported among similar age-group adolescents in Western countries. In this large-scale cross-sectional study, with consideration of sedentary behaviors and other potential confounders, we found a negative relationship between PA and depression that students in the active group were less likely to be depressive than their inactive counterparts. In other words, inactive adolescents were at elevated risk of being depressive compared to their active counterparts.

Most previous studies reported a negative association between physical activity and depression but none of them took into consideration of sedentary behaviors (i.e., time spent on watching TV, course study time and sleeping time) [3-13,15-17]. Our study enabled us to consider time spent on sedentary behavior in our analysis. The PA-depression association remained unchanged after adjustment for sedentary behaviors and other potential confounders, which suggested that sedentary behaviors imposed little influence on the PA-depression association in this sample population.

Several plausible mechanisms for how physical activity affects depression have been proposed. Physical activity may have physiological effects on depression due to an increased release of β-endorphins, brain neurotransmitters (e.g., serotonin, dopamine, and norepinephrine) [33]. Another possible explanation is that exercise reduces emotional strain and serves as a buffer against stressful events. Next, participation in regular physical exercise programs may convey a sense of mastery and increased self-esteem [34-36]. Participation in sport and exercise groups may also provide social interaction and promote participants' social skills. In addition, adolescents participated in after-class physical activity in natural or 'green' environments, typically parks, open spaces, and playgrounds, which would benefit their mental health [37,38].

The negative relationship between physical activity and depression may have implications for primary and secondary prevention of depression in adolescents. Though drugs are a common and recommended modality for the short-term treatment of adolescent depression [39], medications have unwanted side-effects, and the long-term efficacy and safety of antidepressants have not yet been confirmed by large-scale randomized controlled trials. Given an uncertain long-term efficacy of drug therapy and another often used approach, psychotherapy, for treating adolescent depression, it is important to continue to investigate the efficacy of low-risk interventions for reducing depression, such as physical activity, that may be more acceptable to youth and families.

To the best of our knowledge, this is not only the first study regarding relationship between physical activity and depression in Mainland China, but also the first one to reveal a negative association between physical activity time and depression with consideration of sedentary behaviors among high school students. This study could thus be able to add more solid evidence to the current literature on PA-depression association. The sample in this study was randomly selected from urban high-school students in Nanjing, China, with a high response rate. However, there were still several limitations of our study. First, our study did not allow us to infer causality for the depression-PA relationships, because adolescents with depression might be inactive. As the study was cross-sectional, the temporal relationship between adolescent depression and physical activity could not be accurately defined. A prospective study is recommended to further examine the potential causal relationship between physical activity and depression. Ultimately, randomized controlled trials are needed to evaluate the causal relationship. Another major limitation was related to self-reported time spent in physical activity and sedentary behaviors. This may have resulted in some potential bias. However, the self-reporting measurements have demonstrated to have sufficient validity and reliability in epidemiological study, and it has been widely accepted in such public health researches [40-42]. Next, there might be potential clustering effects at class level in the study sample, because the participants were selected using multi-stage cluster (class-based) sampling approach rather than a simple random sampling method. Fourth, disabilities/illness and life events may influence both participants' activity level and the occurrence of depression. However, because such data were unavailable, we could not put them into consideration. We are aware of this as one of the limitations of the current study.

In a developing society like China with rapid social and economic transition to industrialization, students spend more time on academic studies but less time on physical activity compared to their counterparts in developed societies. As a consequence of such 'unhealthy' lifestyles, more and more students are tending to become overweight and depressive, especially in urban areas [27]. Depression is a substantial problem facing young adolescents. The study highlights the need for more intervention at the middle school level. Health promotion strategies and lifestyle interventions targeting students with lower physical activity time in the current context of Mainland China could be helpful in the campaigns against depression.

## Conclusion

Physical activity was negatively associated with depression among Chinese adolescents in Mainland China. Our study has important implications for health officers and public health professionals to pay much attention on association between physical activity and depression in Mainland China.

## Competing interests

The authors declare that they have no competing interests.

## Authors' contributions

XH contributed to data analysis and paper writing. FX (PI of the project) contributed to study design, data collection, and paper writing. JQL (Co-PI of the project) involved in study design, data collection and paper writing. LAT participated in study design, data analysis and paper writing. Both YQL and ZYW took part in study design and data collection and analysis. ITY and SG contributed to manuscript writing and language editing. All authors reviewed drafts of the manuscript and approved the final manuscript.

## Pre-publication history

The pre-publication history for this paper can be accessed here:


